# Changes in intraocular pressure during surgery in the lateral decubitus position under sevoflurane and propofol anesthesia

**DOI:** 10.1007/s10877-015-9787-3

**Published:** 2015-10-06

**Authors:** Makiko Hardy Yamada, Tomonori Takazawa, Nobuhisa Iriuchijima, Tatsuo Horiuchi, Shigeru Saito

**Affiliations:** 1 Department of Anesthesiology, Gunma University Graduate School of Medicine, 3-39-22 Showa-machi, Maebashi, 371-8511 Japan; 2 Department of Anesthesiology, Kiryu Kosei General Hospital, 6-3 Orihime-cho, Kiryu, 376-0024 Japan

**Keywords:** Intraocular pressure, Lateral decubitus position, Sevoflurane, Propofol

## Abstract

Intraocular pressure (IOP) has been shown to change with body position. Several studies have shown that the lateral decubitus position (LDP) is associated with a significant increase in IOP in the dependent eye. However, whether anesthetic agents alter IOP in the LDP remains unclear. This study investigated the effect of sevoflurane and propofol anesthesia on IOP in the LDP. A total of 28 patients undergoing surgery in the LDP were included. Patients were randomly allocated to sevoflurane or propofol groups. IOP in both eyes was recorded and compared between groups at five time points: after anesthesia induction, after endotracheal intubation, at 5 min and 1 h after a positional change to the LDP, and 5 min after returning to the supine position. In the sevoflurane group, IOP was significantly increased in both dependent and non-dependent eyes 1 h after changing to the LDP. In the propofol group, IOP decreased in both dependent and non-dependent eyes after tracheal intubation, but did not increase after changing to the LDP. The number of patients in whom IOP increased to ≥28 mmHg was greater in the sevoflurane group than in the propofol group. Propofol may be better than sevoflurane for the maintenance of anesthesia in the LDP. Monitoring of IOP in the LDP might help avoid ophthalmic complications.

## Introduction

Intraocular pressure (IOP) is influenced by several factors, including changes in body position. For example, IOP is known to increase in the lateral decubitus position (LDP) in both awake healthy subjects and glaucoma patients [1–6]. Recent evidence indicates that increases in IOP are not directly related to the development of ischemic optic neuropathy, which is the most common cause of postoperative visual loss (POVL) [7]. However, increased IOP is likely to be a risk factor for other ophthalmic complications, including anterior ischemic optic neuropathy, retinal artery occlusion, and deterioration of preoperative glaucoma [8]. Therefore, monitoring IOP during general anesthesia may allow anesthesiologists to reduce the risk of perioperative ophthalmic complications.

Previous reports have documented the potential risk of ophthalmic complications during anesthesia in the LDP. One case report describes a 59-year-old woman who suffered complete visual loss in the dependent eye after spine surgery in the LDP [9]. In addition, two cases of POVL in which spine surgery was performed in the LDP are included in the American Society of Anesthesiologists (ASA) POVL Registry [10]. Despite these case reports, the effect of the LDP on IOP during general anesthesia still remains to be elucidated. The sole study addressing this issue demonstrated that IOP was significantly increased, particularly in the dependent eye, in the LDP [11].

Anesthetic agents that are appropriate for management of IOP intraoperatively would be of interest to anesthesiologists. In the supine position, significant reductions in IOP after induction and maintenance of anesthesia with both sevoflurane and propofol have been reported [12], while IOP values during laparoscopic gynecological procedures in the Trendelenburg position under isoflurane anesthesia were higher than those under propofol anesthesia [13]. In addition, the reduction in IOP during cataract surgery in the supine position was significantly more pronounced under propofol anesthesia compared with sevoflurane anesthesia [14]. In contrast, no significant difference in IOP was seen during spine surgery in the prone position under sevoflurane anesthesia compared with propofol anesthesia [15]. Therefore, it remains controversial whether volatile or intravenous anesthetic agents are preferable to avoid abnormal changes in IOP.

Besides IOP, ocular perfusion pressure (OPP) has also been implicated as an important factor in ophthalmic diseases such as glaucoma [4]. OPP is the driving force for ocular blood flow, and low or unstable OPP has been associated with the development or progression of open-angle glaucoma [16]. However, few studies have reported on changes in OPP during surgery in the LDP. The purpose of this study was to investigate the changes in IOP and OPP during surgery in the LDP under sevoflurane and propofol anesthesia.

## Methods

After approval by the human studies committee, written informed consent for participation in the study was obtained from 28 patients scheduled for surgery in the LDP between July 2007 and February 2010. Patients who had glaucoma and ophthalmic disease other than myopia or hypermetropia were excluded. All patients were randomly assigned to either a sevoflurane group (n = 14) or a propofol group (n = 14). In the sevoflurane group, 10 patients had a lung operation, 3 had hip replacement arthroplasty, and 1 had a femoral plate removed. In the propofol group, 11 patients had a lung operation, while three had a nephrectomy. No premedication was given to patients before anesthesia. Anesthesia was induced with 1.8–2.5 mg/kg propofol in the sevoflurane group, and with a target-controlled infusion (TCI) of propofol (3.0–5.0 μg/ml) in the propofol group. Simultaneously, 0.2–0.5 μg/kg/min remifentanil was administered for induction of anesthesia in both groups. Vecuronium (0.12–0.15 mg/kg) or rocuronium (0.65–0.9 mg/kg) was administered to facilitate tracheal intubation. Mechanical ventilation was started and adjusted to maintain the end-tidal CO_2_ (ETCO_2_) partial pressure between 25 and 40 mmHg. A 22-gauge catheter was placed in the radial artery in the dependent arm to monitor arterial pressure. After induction of anesthesia, all patients were turned from the supine position to the LDP. None of the patients adopted a low head position in the LDP. A soft pillow was inserted under the head to maintain the head parallel to the center of the thoracic vertebra. The absence of extraocular pressure from a sponge pillow was confirmed. Anesthesia was maintained with 1.5–2.0 % sevoflurane in the sevoflurane group and with a TCI of propofol (2.8–4 μg/ml) in the propofol group. Intermittent bolus injections of 50-100 μg fentanyl and/or a continuous infusion of 0.1–0.3 μg/kg/min remifentanil were given for maintenance of general anesthesia in both groups. The depth of anesthesia was controlled clinically with the aim to maintain vital signs within ±20 % of the preoperative value. Muscle relaxant agents were used as needed intraoperatively. After completion of the surgical procedure, the patients were moved back to the supine position. Upon arousal from anesthesia, the patients were asked about any vision changes or ocular discomfort.

IOP, mean blood pressure (mBP), heart rate, and ETCO_2_ were recorded at the following five time points: (T1) in the supine position, after induction of anesthesia by propofol, and before injection of the muscle relaxant (defined as control); (T2) after tracheal intubation and just before injection of the muscle relaxant; (T3) 5 min after positioning in the LDP; (T4) 1 h after adoption of the LDP; and (T5) 5 min after changing back to the supine position.

IOP was measured with Tono-Pen^®^ XL Applanation tonometer (Reichert, Depew, NY, USA). This instrument measures and calculates IOP by the principle of the Imbert–Fick law (P = F/A, where P = IOP, F = the force exerted by the tonometer to flatten a specific area of the eye, and A = the area flattened). Each IOP datum was obtained by the calculated average of three successful tonometer measurements, the range of difference of which was <10 %. The Tono-Pen was calibrated before measurements. IOP of the right eye was always measured first. OPP was calculated according to the formula: OPP = 115/130 × mBP − IOP [4].

### Statistical analysis

Sample size was calculated using power analysis. A pilot study of 10 patients revealed that the average value and standard deviation of IOP measured in the eyes in the LDP were 19.6 and 4.9 mmHg, respectively. We assumed that the difference in mean IOP values between both groups was 3.8 mmHg [11]. At the 0.05 level (= α) with a power (1 − β) of 0.8, we calculated that the study required a minimum of 14 patients. Data are expressed as mean ± SD. Mann–Whitney U test was used for between-group comparisons. Categorical data were compared using Fisher’s exact test. The correlation coefficient between IOP and mBP/ETCO_2_ was calculated. Comparisons of measured variables, such as hemodynamic and IOP values, were made using two-way factorial analysis of variance (ANOVA) with repeated measures, followed by the Student–Newmann–Keuls test for multiple comparisons. *P* < 0.05 was considered a significant difference.

## Results

No complications related to IOP measurements were noted intraoperatively or postoperatively. Table 1 shows the demographic variables of patients in the sevoflurane and propofol groups. There was no difference in age, sex, height, weight, body mass index (BMI), preoperative value of hemoglobin, and pre-existing comorbidity, including hypertension and diabetes mellitus. None of the patients had hyperlipidemia. Table 2 shows the intraoperative variables in each group. There were also no significant differences in the duration of anesthesia, operation, and LDP, nor were there differences in the total amount of blood loss and urine output. Table 3 shows the hemodynamic variables and ETCO_2_. No difference in these values was identified between the two groups at any time point. No associations were evident between IOP values and ETCO_2_ in either the sevoflurane group (dependent eye: r^2^ = 0.006, *P* = 0.55, non-dependent eye: r^2^ = 0.04, *P* = 0.12) or the propofol group (dependent eye: r^2^ = 0.05, *P* = 0.08, non-dependent eye: r^2^ = 0.05, *P* = 0.07). Moreover, there was no association between IOP values and mBP in either the sevoflurane group (dependent eye: r^2^ = 0.02, *P* = 0.30, non-dependent eye: r^2^ = 0.02, *P* = 0.78) or the propofol group (dependent eye: r^2^ = 0.02, *P* = 0.27, non-dependent eye: r^2^ = 0.05, *P* = 0.06).

**Table 1:** Demographic data of patients

	Sevoflurane (n = 14)	Propofol (n = 14)	*P*
Age (year)	63.5 ± 16.0	66.1 ± 7.5	0.58
Male	7	6	0.73
Height (cm)	156.7 ± 8.3	157.3 ± 7.7	0.80
Weight (kg)	52.7 ± 9.9	56.4 ± 10.1	0.73
BMI	21.3 ± 2.6	22.7 ± 3.0	0.21
Hemoglobin(g/dl)	12.1 ± 1.6	12.7 ± 1.9	0.40
Hypertension	5	9	0.16
(W. medication)	4	6	0.47
Diabetes mellitus	2	1	0.61
(W. medication)	1	1	1.00
Lung Operation	10	11	0.69
Other Operation	4	3	

Data are shown as mean ± SD or as the number. Statistical analyses were performed using the Mann–Whitney U test or Fisher’s exact test

**Table 2:** Intraoperative data

	Sevoflurane (n = 14)	Propofol (n = 14)	*P*
Anesthesia time (min)	255 ± 61	301 ± 93	0.18
Operation time (min)	189 ± 60	242 ± 93	0.11
Duration of prone position (min)	214 ± 61	267 ± 90	0.13
Blood loss (mL)	666 ± 1744	143 ± 193	0.29
Urine output (mL)	697 ± 568	681 ± 696	0.68

Data are shown as mean ± SD. Statistical analyses were performed by the Mann–Whitney U test

**Table 3:** Hemodynamic variables and ETCO_2_

	Sevoflurane (n = 14)	Propofol (n = 14)	*P*
mBP (mmHg)			
T1: control	87 ± 9	87 ± 21	0.74
T2: intubation	75 ± 13	70 ± 12	0.42
T3: lateral	79 ± 17	71 ± 14	0.16
T4: lateral 1 h	76 ± 12	78 ± 13	0.68
T5: supine	82 ± 13	79 ± 17	0.63
Heart rate (bpm)			
T1	75 ± 23	72 ± 13	0.64
T2	68 ± 22	71 ± 14	0.61
T3	67 ± 17	71 ± 14	0.52
T4	72 ± 14	77 ± 11	0.43
T5	73 ± 15	75 ± 11	0.68
ETCO_2_ (mmHg)			
T1	27 ± 6	31 ± 6	0.07
T2	32 ± 6	34 ± 6	0.26
T3	36 ± 3	32 ± 6	0.07
T4	34 ± 5	34 ± 6	0.81
T5	34 ± 6	34 ± 6	0.87

Data are shown as mean ± SD. There was no difference in mBP, heart rate and ETCO_2_ between the sevoflurane and propofol groups at any time point

**Fig. 1:**
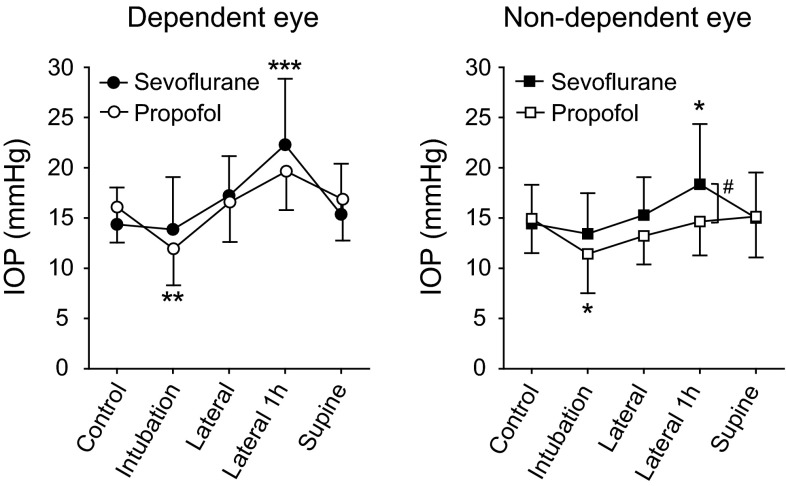
Changes in intraocular pressure (IOP) under sevoflurane and propofol anesthesia *Open and solid circles* indicate IOP measured in the dependent eye under sevoflurane and propofol anesthesia, respectively. *Open and solid squares* indicate IOP measured in the non-dependent eye under sevoflurane and propofol anesthesia, respectively. *P < 0.05. **P < 0.01, ***P < 0.001 versus control within a group. ^#^ *P* < 0.05 between sevoflurane and propofol groups

Figure 1 shows the IOP changes in the dependent and non-dependent eye in each group. In the sevoflurane group, the IOP values in the dependent eye measured 1 h after LDP were greater than control values (One-way ANOVA post hoc Student–Newman–Keuls test, *P* < 0.001). In addition, IOP values in the non-dependent eye measured 1 h after LDP were greater than control values (*P* < 0.05). In contrast, these changes were not observed in the propofol group. Interestingly, in the propofol group, IOP values after tracheal intubation in both dependent and non-dependent eyes were smaller than control values (*P* < 0.05). IOP values in the independent eye measured 1 h after LDP were significantly greater in the sevoflurane group than in the propofol group (Table 4 (*P* < 0.05). Moreover, the number of patients exhibiting an IOP of greater than or equal to 28 mmHg, measured 1 h after LDP, were greater in the sevoflurane group than in the propofol group, Fisher’s exact test, *P* < 0.05). No difference in OPP values of dependent or non-dependent eyes were seen between sevoflurane and propofol groups at any time point (Table 4). Fortunately, no patients in either group experienced ophthalmic complications, such as visual loss or visual field constriction, postoperatively (Table 5).

**Table 4:** IOP and OPP values

	Sevoflurane (n = 14)	Propofol (n = 14)	*P*
IOP of dependent eye (mmHg)			
T1: control	14.4 ± 3.7	16.0 ± 3.5	0.31
T2: intubation	13.9 ± 5.2	11.9 ± 3.6	0.25
T3: lateral	17.2 ± 3.9	16.6 ± 4.0	0.70
T4: lateral 1 h	22.3 ± 6.6	19.6 ± 3.9	0.12
T5: supine	15.4 ± 5.0	16.9 ± 4.1	0.38
IOP of independent eye (mmHg)			
T1	14.4 ± 3.9	14.9 ± 3.4	0.75
T2	13.4 ± 4.0	11.4 ± 4.0	0.20
T3	15.3 ± 3.8	13.2 ± 2.8	0.18
T4	18.4 ± 6.0	14.6 ± 3.4	0.02
T5	15.0 ± 4.5	15.1 ± 4.0	0.93
OPP of dependent eye (mmHg)			
T1	62.5 ± 8.7	62.3 ± 18.0	0.98
T2	52.4 ± 10.9	50.4 ± 10.1	0.68
T3	52.5 ± 13.3	46.3 ± 13.1	0.21
T4	44.5 ± 11.1	49.1 ± 13.7	0.35
T5	57.1 ± 10.3	53.3 ± 14.3	0.43
OPP of independent eye (mmHg)			
T1	62.4 ± 9.6	63.5 ± 17.8	0.83
T2	52.8 ± 12.2	50.9 ± 10.0	0.70
T3	54.4 ± 15.6	49.7 ± 13.2	0.34
T4	48.4 ± 12.1	54.1 ± 13.3	0.26
T5	57.5 ± 12.0	55.0 ± 13.7	0.62

Data are shown as mean ± SD

**Table 5:** Number of patients exhibiting high IOP

Anesthetics	No. of Patients		Fisher’s exact test *P* value
	IOP ≥ 28	IOP < 28	
Sevoflurane	5	9	0.04
Propofol	0	14	

## Discussion

This study showed that IOP values increase in the LDP under sevoflurane but not propofol anesthesia. Although there was no statistically significant difference in IOP values between sevoflurane and propofol groups, an abnormal increase in IOP (i.e. ≥28 mmHg) in the LDP was more prominent in the sevoflurane group than in the propofol group. This evidence suggests that propofol may be better than sevoflurane for maintenance of anesthesia during surgery performed in the LDP.

One important finding of this study was the increase in IOP in the LDP under sevoflurane anesthesia. This was consistent with a previous report [11]. In contrast, an increase in IOP in the LDP was not observed under propofol anesthesia. These distinct effects of anesthetics on IOP may be explained as follows. It is postulated that change in body position from supine to lateral increased IOP. Increase in choroidal vascular volume and episcleral venous pressure may play an important role in the increase in IOP in the LDP as well as prone position [11]. Several previous studies, as well as our results, indicate that propofol itself appears to decrease IOP [13, 14, 17]. While the reducing effect of propofol on IOP may mask the increasing effect of LDP on IOP, the increasing effect of LDP may be dominant under sevoflurane anesthesia. The mechanisms of how anesthetics directly affect IOP are not known. Therefore, further studies are required to clarify this issue.

A past study suggested a correlation between PaCO_2_ and IOP in patients who underwent laparoscopic surgery under halothane and N_2_O anesthesia [18]. In the current study, we investigated the relationship between IOP and ETCO_2_ instead of PaCO_2_. There was no correlation between IOP and ETCO_2_. This result was probably due to control of ETCO_2_ within a relatively narrower range compared to the past study (25–40 vs. 15–90 mmHg, respectively). We also examined the relationship between IOP and mBP, which was maintained within ±20 % of the preoperative value. There was no correlation between mBP and IOP, which is consistent with a past report [19]. As shown in Table 3, there was no difference in hemodynamic variables and ETCO_2_ between the sevoflurane and propofol groups. Taken together, the distinct effect of anesthetics on IOP is likely to result from the anesthetics per se rather than their secondary effect on hemodynamic variables.

We reported here that OPP values did not differ between the sevoflurane and propofol groups. This result suggests that these anesthetic agents show little difference in maintaining an appropriate OPP. This may simply reflect the lack of difference in mBP, which is directly related to OPP, between groups. Holding an adequate OPP during surgery in the LDP is likely to be important to prevent development or progression of ischemic optic neuropathy or glaucoma. However, the appropriate range of OPPs for which anesthesiologists should aim during surgery remains unclear. Further study is needed to clarify the relationship between control of OPP during surgery and occurrence of POVL after surgery.

One of the limitations of the current study was the lack of depth-of-anesthesia monitoring. Depth of anesthesia was controlled based on standard clinical monitoring practices. None of the patients complained of intraoperative awareness. We believe that there was no significant difference in the depth of anesthesia, because hemodynamic variables did not differ between the groups at any time point. However, some objective measures, such as BIS value, should be monitored to evaluate depth of anesthesia. Another limitation is the heterogeneous population of subjects. Although nearly 80 % of patients in both groups underwent lung operations, the remaining patients underwent various surgeries, including nephrectomy and lower limb surgery. The ratio of lung to other operations in both groups was not different, as shown in Table 1. Therefore, we believe that the effect of the heterogeneous population of subjects on the changes in IOP in the LDP would be negligible. Based on the self-reported history, patients who had glaucoma and ophthalmic disease other than myopia or hypermetropia were excluded. The maximum IOP measured at T1 in both groups was 21 mmHg. Normal IOP in the Japanese population is reportedly between 10 and 21 mmHg [20]. Taken together, we believe that no patients with glaucoma and high IOP were included in this study. However, we did not perform comprehensive ophthalmic examinations before the operation, so we cannot exclude the possibility that patients with mild to moderate glaucoma or ocular hypertension may have been included. IOP measurements should have been performed by observers blinded to study groupings, but were not in this study.

Risk factors of POVL include male sex, obesity, use of the Wilson spinal frame, longer anesthesia duration, greater blood loss, and a lower percentage of colloid in the non-blood fluid administered [7]. Some of these risk factors appear to be common for other ophthalmic complications after operations performed in the LDP. In our study, none of the patients complained of ophthalmic complications despite the abnormal increase in IOP in some of the patients. Patients with pre-existing ophthalmic conditions, including glaucoma, were excluded from the current study. Moreover, patients with a high risk of POVL, such as obesity and long operation time, were not included. These factors might have contributed to the absence of ophthalmic complications. The current study suggested that physicians should be cautious when patients undergo surgery in the LDP.
